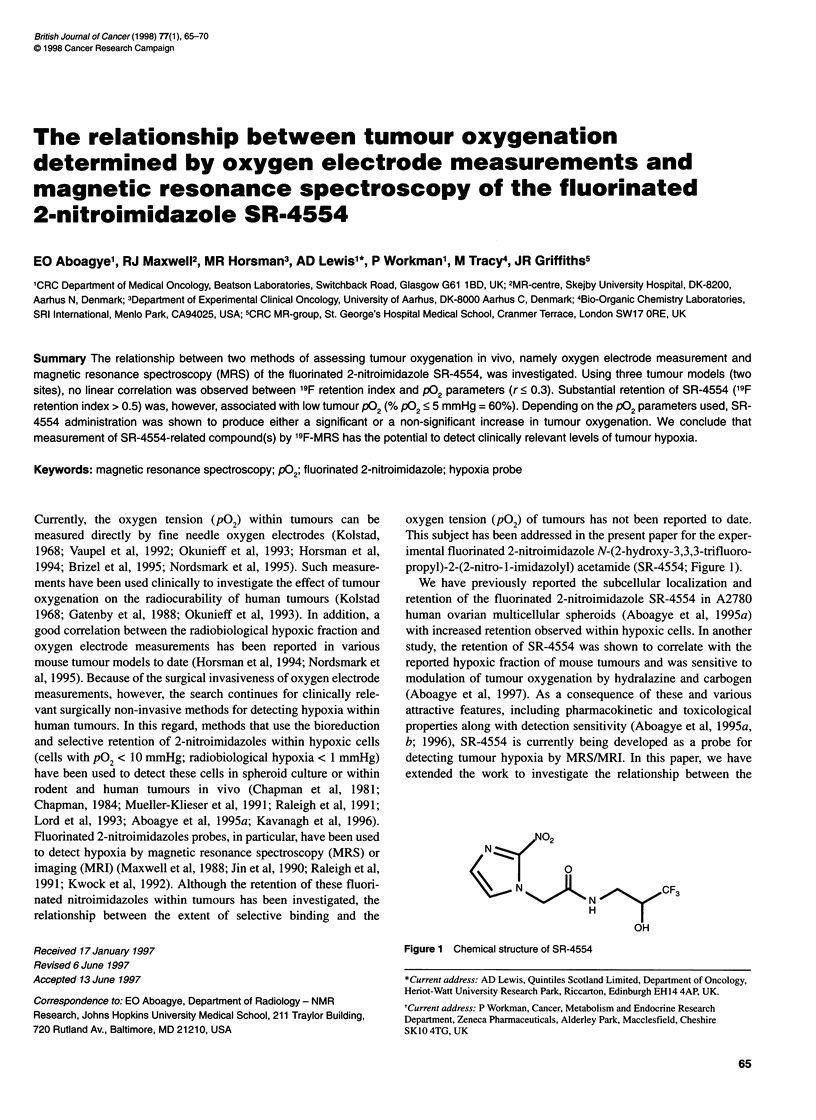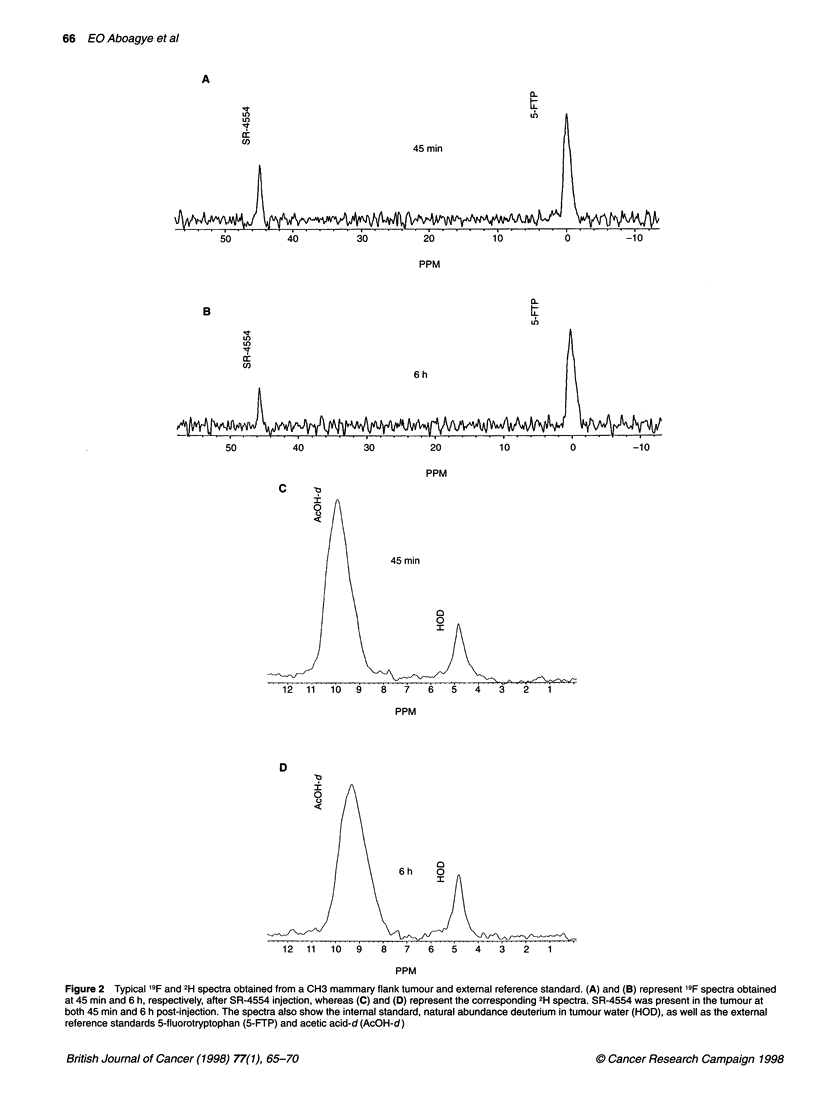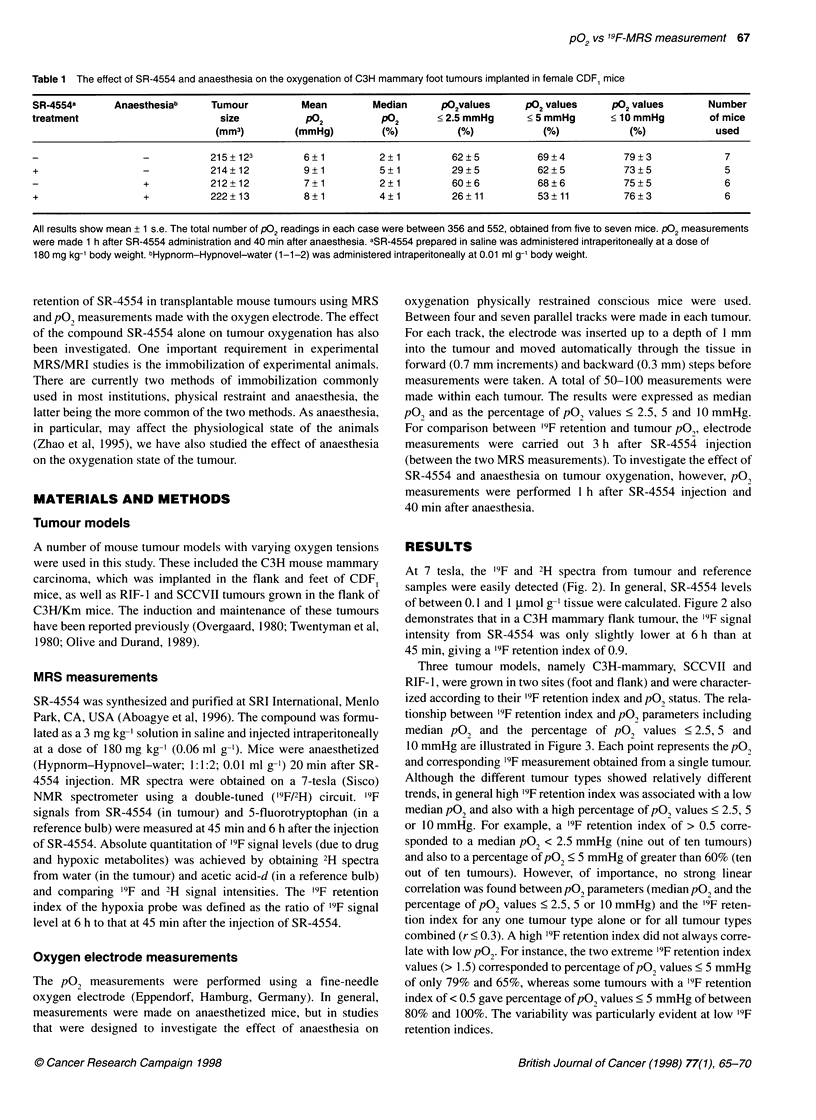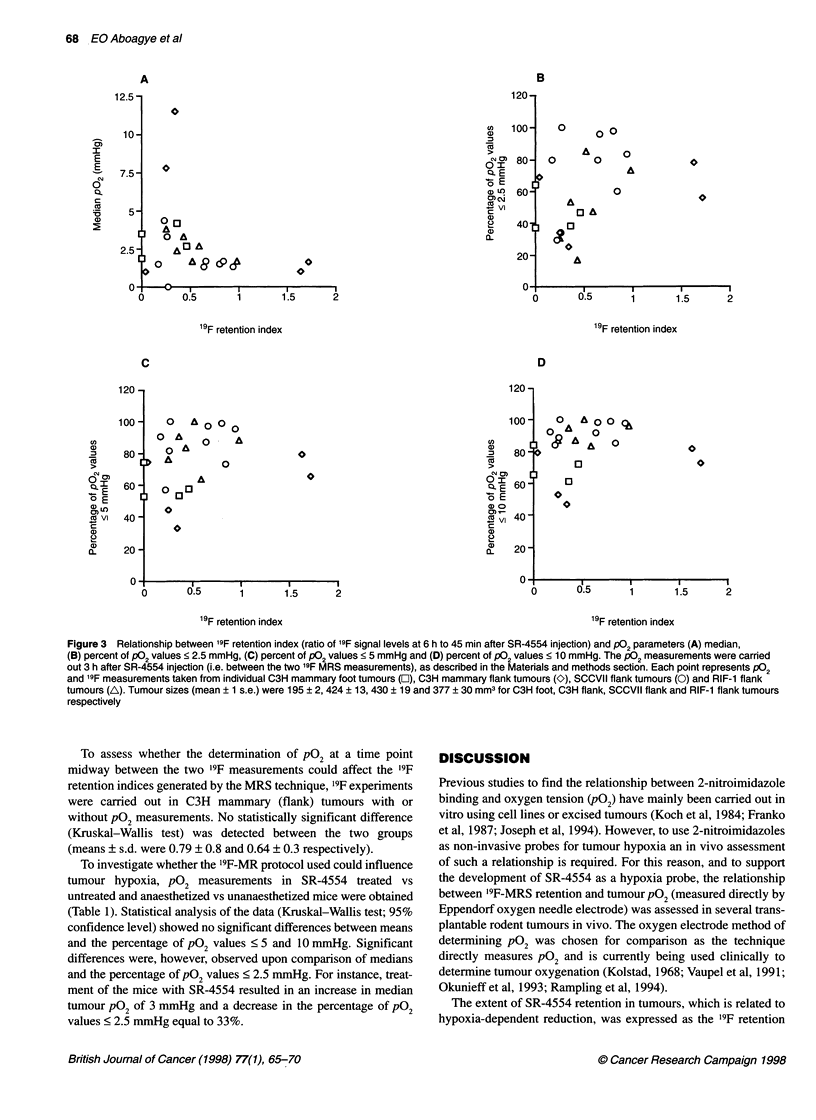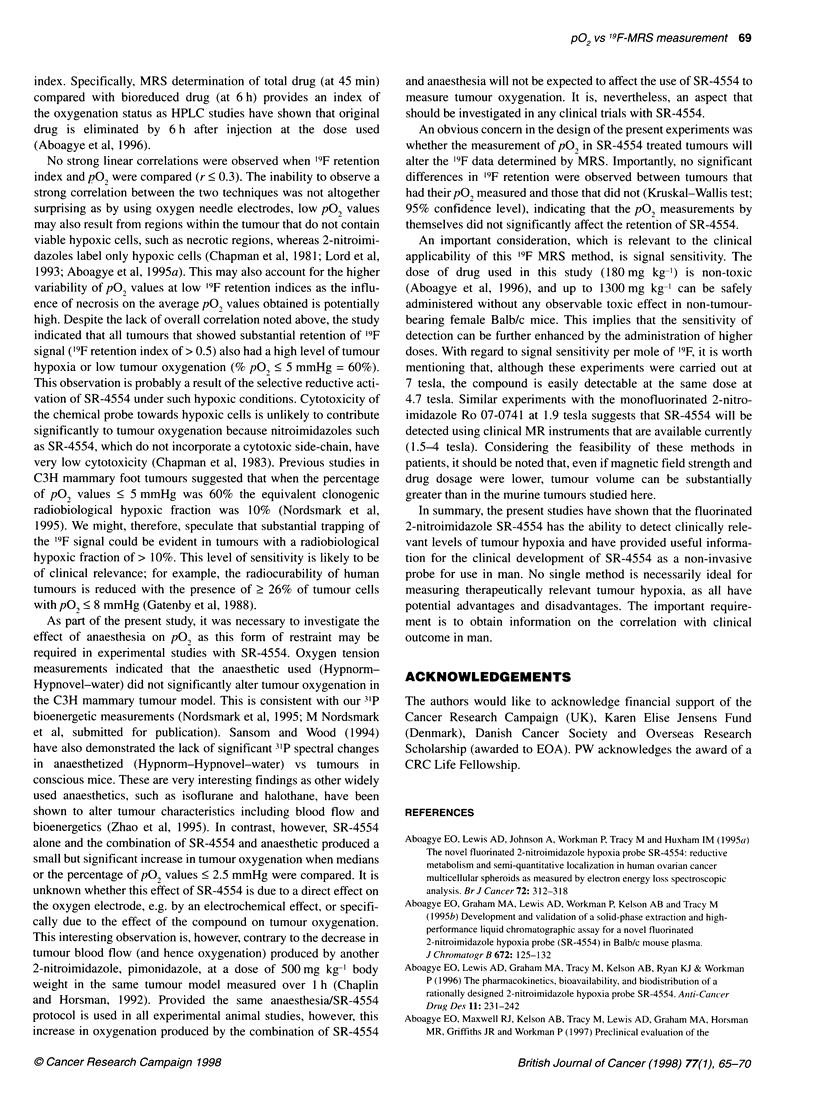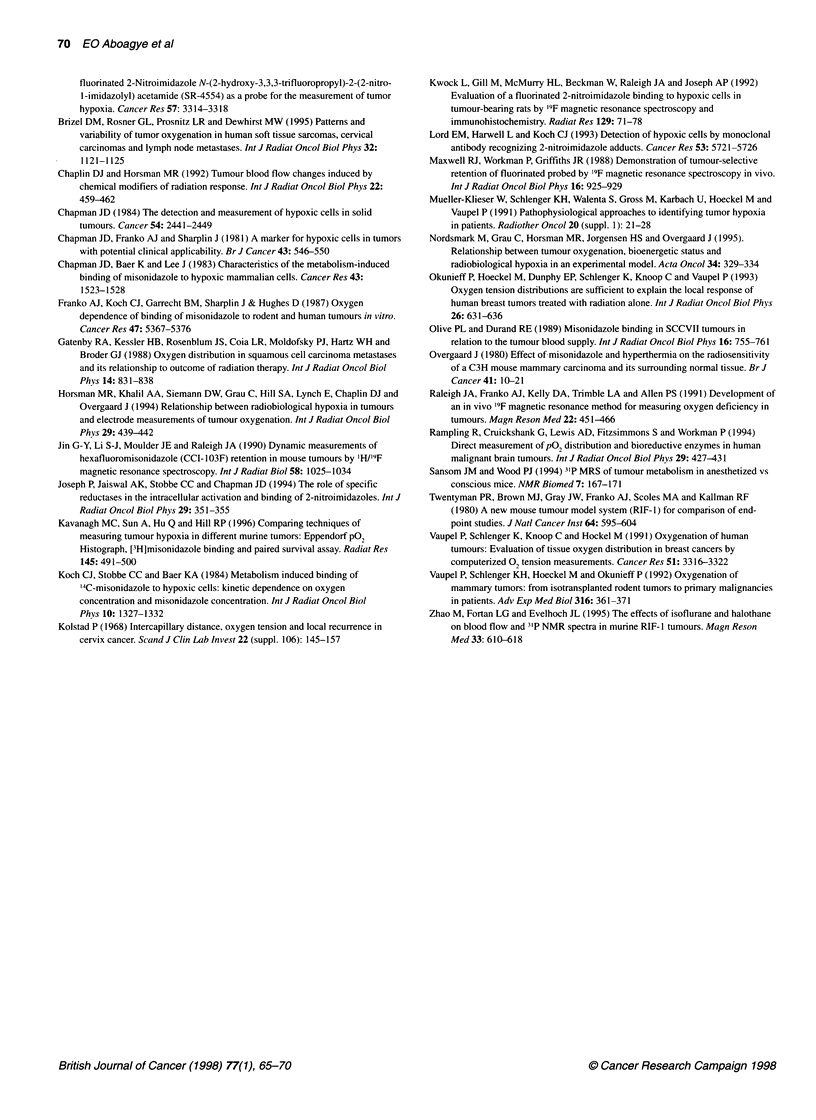# The relationship between tumour oxygenation determined by oxygen electrode measurements and magnetic resonance spectroscopy of the fluorinated 2-nitroimidazole SR-4554.

**DOI:** 10.1038/bjc.1998.10

**Published:** 1998

**Authors:** E. O. Aboagye, R. J. Maxwell, M. R. Horsman, A. D. Lewis, P. Workman, M. Tracy, J. R. Griffiths

**Affiliations:** CRC Department of Medical Oncology, Beatson Laboratories, Glasgow, UK.

## Abstract

The relationship between two methods of assessing tumour oxygenation in vivo, namely oxygen electrode measurement and magnetic resonance spectroscopy (MRS) of the fluorinated 2-nitroimidazole SR-4554, was investigated. Using three tumour models (two sites), no linear correlation was observed between 19F retention index and pO2 parameters (r < or = 0.3). Substantial retention of SR-4554 (19F retention index > 0.5) was, however, associated with low tumour pO2 (% pO2 < or = 5 mmHg = 60%). Depending on the pO2 parameters used, SR-4554 administration was shown to produce either a significant or a non-significant increase in tumour oxygenation. We conclude that measurement of SR-4554-related compound(s) by 19F-MRS has the potential to detect clinically relevant levels of tumour hypoxia.


British Journal of Cancer (1998) 77(1), 65–70
© 1998 Cancer Research Campaign

### The relationship between tumour oxygenation determined by oxygen electrode measurements and magnetic resonance spectroscopy of the fluorinated 2-nitroimidazole SR-4554

**EO Aboagye[1], RJ Maxwell[2], MR Horsman[3], AD Lewis[1*], P Workman[1], M Tracy[4], JR Griffiths[5]**

[1]CRC Department of Medical Oncology, Beatson Laboratories, Switchback Road, Glasgow G61 1BD, UK; [2]MR-centre, Skejby University Hospital, DK-8200, Aarhus N, Denmark; [3]Department of Experimental Clinical Oncology, University of Aarhus, DK-8000 Aarhus C, Denmark; [4]Bio-Organic Chemistry Laboratories, SRI International, Menlo Park, CA94025, USA; [5]CRC MR-group, St. George's Hospital Medical School, Cranmer Terrace, London SW17 0RE, UK

**Summary** The relationship between two methods of assessing tumour oxygenation in vivo, namely oxygen electrode measurement and magnetic resonance spectroscopy (MRS) of the fluorinated 2-nitroimidazole SR-4554, was investigated. Using three tumour models (two sites), no linear correlation was observed between $^{19}F$ retention index and $pO_2$ parameters ($r \leq 0.3$). Substantial retention of SR-4554 ($^{19}F$ retention index > 0.5) was, however, associated with low tumour $pO_2$ (% $pO_2 \leq 5$ mmHg = 60%). Depending on the $pO_2$ parameters used, SR-4554 administration was shown to produce either a significant or a non-significant increase in tumour oxygenation. We conclude that measurement of SR-4554-related compound(s) by $^{19}F$-MRS has the potential to detect clinically relevant levels of tumour hypoxia.

**Keywords:** magnetic resonance spectroscopy; $pO_2$; fluorinated 2-nitroimidazole; hypoxia probe

Currently, the oxygen tension ($pO_2$) within tumours can be measured directly by fine needle oxygen electrodes (Kolstad, 1968; Vaupel et al, 1992; Okunieff et al, 1993; Horsman et al, 1994; Brizel et al, 1995; Nordsmark et al, 1995). Such measurements have been used clinically to investigate the effect of tumour oxygenation on the radiocurability of human tumours (Kolstad 1968; Gatenby et al, 1988; Okunieff et al, 1993). In addition, a good correlation between the radiobiological hypoxic fraction and oxygen electrode measurements has been reported in various mouse tumour models to date (Horsman et al, 1994; Nordsmark et al, 1995). Because of the surgical invasiveness of oxygen electrode measurements, however, the search continues for clinically relevant surgically non-invasive methods for detecting hypoxia within human tumours. In this regard, methods that use the bioreduction and selective retention of 2-nitroimidazoles within hypoxic cells (cells with $pO_2$ < 10 mmHg; radiobiological hypoxia < 1 mmHg) have been used to detect these cells in spheroid culture or within rodent and human tumours in vivo (Chapman et al, 1981; Chapman, 1984; Mueller-Klieser et al, 1991; Raleigh et al, 1991; Lord et al, 1993; Aboagye et al, 1995*a*; Kavanagh et al, 1996). Fluorinated 2-nitroimidazoles probes, in particular, have been used to detect hypoxia by magnetic resonance spectroscopy (MRS) or imaging (MRI) (Maxwell et al, 1988; Jin et al, 1990; Raleigh et al, 1991; Kwock et al, 1992). Although the retention of these fluorinated nitroimidazoles within tumours has been investigated, the relationship between the extent of selective binding and the oxygen tension ($pO_2$) of tumours has not been reported to date. This subject has been addressed in the present paper for the experimental fluorinated 2-nitroimidazole *N*-(2-hydroxy-3,3,3-trifluoropropyl)-2-(2-nitro-1-imidazolyl) acetamide (SR-4554; Figure 1).

We have previously reported the subcellular localization and retention of the fluorinated 2-nitroimidazole SR-4554 in A2780 human ovarian multicellular spheroids (Aboagye et al, 1995*a*) with increased retention observed within hypoxic cells. In another study, the retention of SR-4554 was shown to correlate with the reported hypoxic fraction of mouse tumours and was sensitive to modulation of tumour oxygenation by hydralazine and carbogen (Aboagye et al, 1997). As a consequence of these and various attractive features, including pharmacokinetic and toxicological properties along with detection sensitivity (Aboagye et al, 1995*a*, *b*; 1996), SR-4554 is currently being developed as a probe for detecting tumour hypoxia by MRS/MRI. In this paper, we have extended the work to investigate the relationship between the

**Figure 1** Chemical structure of SR-4554

*Received 17 January 1997*
*Revised 6 June 1997*
*Accepted 13 June 1997*

*Correspondence to:* EO Aboagye, Department of Radiology – NMR Research, Johns Hopkins University Medical School, 211 Traylor Building, 720 Rutland Av., Baltimore, MD 21210, USA

**Current address:* AD Lewis, Quintiles Scotland Limited, Department of Oncology, Heriot-Watt University Research Park, Riccarton, Edinburgh EH14 4AP, UK.

†*Current address:* P Workman, Cancer, Metabolism and Endocrine Research Department, Zeneca Pharmaceuticals, Alderley Park, Macclesfield, Cheshire SK10 4TG, UK

**Figure 2** Typical $^{19}F$ and $^{2}H$ spectra obtained from a CH3 mammary flank tumour and external reference standard. (**A**) and (**B**) represent $^{19}F$ spectra obtained at 45 min and 6 h, respectively, after SR-4554 injection, whereas (**C**) and (**D**) represent the corresponding $^{2}H$ spectra. SR-4554 was present in the tumour at both 45 min and 6 h post-injection. The spectra also show the internal standard, natural abundance deuterium in tumour water (HOD), as well as the external reference standards 5-fluorotryptophan (5-FTP) and acetic acid-*d* (AcOH-*d*)

*© Cancer Research Campaign 1998*

**Table 1** The effect of SR-4554 and anaesthesia on the oxygenation of C3H mammary foot tumours implanted in female $CDF_1$ mice

| SR-4554[a] treatment | Anaesthesia[b] | Tumour size ($mm^3$) | Mean $pO_2$ (mmHg) | Median $pO_2$ (%) | $pO_2$ values ≤ 2.5 mmHg (%) | $pO_2$ values ≤ 5 mmHg (%) | $pO_2$ values ≤ 10 mmHg (%) | Number of mice used |
|---|---|---|---|---|---|---|---|---|
| – | – | 215 ± 12³ | 6 ± 1 | 2 ± 1 | 62 ± 5 | 69 ± 4 | 79 ± 3 | 7 |
| + | – | 214 ± 12 | 9 ± 1 | 5 ± 1 | 29 ± 5 | 62 ± 5 | 73 ± 5 | 5 |
| – | + | 212 ± 12 | 7 ± 1 | 2 ± 1 | 60 ± 6 | 68 ± 6 | 75 ± 5 | 6 |
| + | + | 222 ± 13 | 8 ± 1 | 4 ± 1 | 26 ± 11 | 53 ± 11 | 76 ± 3 | 6 |

All results show mean ± 1 s.e. The total number of $pO_2$ readings in each case were between 356 and 552, obtained from five to seven mice. $pO_2$ measurements were made 1 h after SR-4554 administration and 40 min after anaesthesia. [a]SR-4554 prepared in saline was administered intraperitoneally at a dose of 180 mg $kg^{-1}$ body weight. [b]Hypnorm–Hypnovel–water (1–1–2) was administered intraperitoneally at 0.01 ml $g^{-1}$ body weight.

retention of SR-4554 in transplantable mouse tumours using MRS and $pO_2$ measurements made with the oxygen electrode. The effect of the compound SR-4554 alone on tumour oxygenation has also been investigated. One important requirement in experimental MRS/MRI studies is the immobilization of experimental animals. There are currently two methods of immobilization commonly used in most institutions, physical restraint and anaesthesia, the latter being the more common of the two methods. As anaesthesia, in particular, may affect the physiological state of the animals (Zhao et al, 1995), we have also studied the effect of anaesthesia on the oxygenation state of the tumour.

### MATERIALS AND METHODS

### Tumour models

A number of mouse tumour models with varying oxygen tensions were used in this study. These included the C3H mouse mammary carcinoma, which was implanted in the flank and feet of $CDF_1$ mice, as well as RIF-1 and SCCVII tumours grown in the flank of C3H/Km mice. The induction and maintenance of these tumours have been reported previously (Overgaard, 1980; Twentyman et al, 1980; Olive and Durand, 1989).

### MRS measurements

SR-4554 was synthesized and purified at SRI International, Menlo Park, CA, USA (Aboagye et al, 1996). The compound was formulated as a 3 mg $kg^{-1}$ solution in saline and injected intraperitoneally at a dose of 180 mg $kg^{-1}$ (0.06 ml $g^{-1}$). Mice were anaesthetized (Hypnorm–Hypnovel–water; 1:1:2; 0.01 ml $g^{-1}$) 20 min after SR-4554 injection. MR spectra were obtained on a 7-tesla (Sisco) NMR spectrometer using a double-tuned ($^{19}F/^{2}H$) circuit. $^{19}F$ signals from SR-4554 (in tumour) and 5-fluorotryptophan (in a reference bulb) were measured at 45 min and 6 h after the injection of SR-4554. Absolute quantitation of $^{19}F$ signal levels (due to drug and hypoxic metabolites) was achieved by obtaining $^{2}H$ spectra from water (in the tumour) and acetic acid-*d* (in a reference bulb) and comparing $^{19}F$ and $^{2}H$ signal intensities. The $^{19}F$ retention index of the hypoxia probe was defined as the ratio of $^{19}F$ signal level at 6 h to that at 45 min after the injection of SR-4554.

### Oxygen electrode measurements

The $pO_2$ measurements were performed using a fine-needle oxygen electrode (Eppendorf, Hamburg, Germany). In general, measurements were made on anaesthetized mice, but in studies that were designed to investigate the effect of anaesthesia on oxygenation physically restrained conscious mice were used. Between four and seven parallel tracks were made in each tumour. For each track, the electrode was inserted up to a depth of 1 mm into the tumour and moved automatically through the tissue in forward (0.7 mm increments) and backward (0.3 mm) steps before measurements were taken. A total of 50–100 measurements were made within each tumour. The results were expressed as median $pO_2$ and as the percentage of $pO_2$ values ≤ 2.5, 5 and 10 mmHg. For comparison between $^{19}F$ retention and tumour $pO_2$, electrode measurements were carried out 3 h after SR-4554 injection (between the two MRS measurements). To investigate the effect of SR-4554 and anaesthesia on tumour oxygenation, however, $pO_2$ measurements were performed 1 h after SR-4554 injection and 40 min after anaesthesia.

### RESULTS

At 7 tesla, the $^{19}F$ and $^{2}H$ spectra from tumour and reference samples were easily detected (Fig. 2). In general, SR-4554 levels of between 0.1 and 1 μmol $g^{-1}$ tissue were calculated. Figure 2 also demonstrates that in a C3H mammary flank tumour, the $^{19}F$ signal intensity from SR-4554 was only slightly lower at 6 h than at 45 min, giving a $^{19}F$ retention index of 0.9.

Three tumour models, namely C3H-mammary, SCCVII and RIF-1, were grown in two sites (foot and flank) and were characterized according to their $^{19}F$ retention index and $pO_2$ status. The relationship between $^{19}F$ retention index and $pO_2$ parameters including median $pO_2$ and the percentage of $pO_2$ values ≤ 2.5, 5 and 10 mmHg are illustrated in Figure 3. Each point represents the $pO_2$ and corresponding $^{19}F$ measurement obtained from a single tumour. Although the different tumour types showed relatively different trends, in general high $^{19}F$ retention index was associated with a low median $pO_2$ and also with a high percentage of $pO_2$ values ≤ 2.5, 5 or 10 mmHg. For example, a $^{19}F$ retention index of > 0.5 corresponded to a median $pO_2$ < 2.5 mmHg (nine out of ten tumours) and also to a percentage of $pO_2$ ≤ 5 mmHg of greater than 60% (ten out of ten tumours). However, of importance, no strong linear correlation was found between $pO_2$ parameters (median $pO_2$ and the percentage of $pO_2$ values ≤ 2.5, 5 or 10 mmHg) and the $^{19}F$ retention index for any one tumour type alone or for all tumour types combined ($r \leq 0.3$). A high $^{19}F$ retention index did not always correlate with low $pO_2$. For instance, the two extreme $^{19}F$ retention index values (> 1.5) corresponded to percentage of $pO_2$ values ≤ 5 mmHg of only 79% and 65%, whereas some tumours with a $^{19}F$ retention index of < 0.5 gave percentage of $pO_2$ values ≤ 5 mmHg of between 80% and 100%. The variability was particularly evident at low $^{19}F$ retention indices.

**Figure 3** Relationship between $^{19}$F retention index (ratio of $^{19}$F signal levels at 6 h to 45 min after SR-4554 injection) and $pO_2$ parameters (**A**) median, (**B**) percent of $pO_2$ values ≤ 2.5 mmHg, (**C**) percent of $pO_2$ values ≤ 5 mmHg and (**D**) percent of $pO_2$ values ≤ 10 mmHg. The $pO_2$ measurements were carried out 3 h after SR-4554 injection (i.e. between the two $^{19}$F MRS measurements), as described in the Materials and methods section. Each point represents $pO_2$ and $^{19}$F measurements taken from individual C3H mammary foot tumours (□), C3H mammary flank tumours (◇), SCCVII flank tumours (○) and RIF-1 flank tumours (△). Tumour sizes (mean ± 1 s.e.) were 195 ± 2, 424 ± 13, 430 ± 19 and 377 ± 30 mm$^3$ for C3H foot, C3H flank, SCCVII flank and RIF-1 flank tumours respectively

To assess whether the determination of $pO_2$ at a time point midway between the two $^{19}$F measurements could affect the $^{19}$F retention indices generated by the MRS technique, $^{19}$F experiments were carried out in C3H mammary (flank) tumours with or without $pO_2$ measurements. No statistically significant difference (Kruskal–Wallis test) was detected between the two groups (means ± s.d. were 0.79 ± 0.8 and 0.64 ± 0.3 respectively).

To investigate whether the $^{19}$F-MR protocol used could influence tumour hypoxia, $pO_2$ measurements in SR-4554 treated vs untreated and anaesthetized vs unanaesthetized mice were obtained (Table 1). Statistical analysis of the data (Kruskal–Wallis test; 95% confidence level) showed no significant differences between means and the percentage of $pO_2$ values ≤ 5 and 10 mmHg. Significant differences were, however, observed upon comparison of medians and the percentage of $pO_2$ values ≤ 2.5 mmHg. For instance, treatment of the mice with SR-4554 resulted in an increase in median tumour $pO_2$ of 3 mmHg and a decrease in the percentage of $pO_2$ values ≤ 2.5 mmHg equal to 33%.

### DISCUSSION

Previous studies to find the relationship between 2-nitroimidazole binding and oxygen tension ($pO_2$) have mainly been carried out in vitro using cell lines or excised tumours (Koch et al, 1984; Franko et al, 1987; Joseph et al, 1994). However, to use 2-nitroimidazoles as non-invasive probes for tumour hypoxia an in vivo assessment of such a relationship is required. For this reason, and to support the development of SR-4554 as a hypoxia probe, the relationship between $^{19}$F-MRS retention and tumour $pO_2$ (measured directly by Eppendorf oxygen needle electrode) was assessed in several transplantable rodent tumours in vivo. The oxygen electrode method of determining $pO_2$ was chosen for comparison as the technique directly measures $pO_2$ and is currently being used clinically to determine tumour oxygenation (Kolstad, 1968; Vaupel et al, 1991; Okunieff et al, 1993; Rampling et al, 1994).

The extent of SR-4554 retention in tumours, which is related to hypoxia-dependent reduction, was expressed as the $^{19}$F retention

© Cancer Research Campaign 1998

index. Specifically, MRS determination of total drug (at 45 min) compared with bioreduced drug (at 6 h) provides an index of the oxygenation status as HPLC studies have shown that original drug is eliminated by 6 h after injection at the dose used (Aboagye et al, 1996).

No strong linear correlations were observed when $^{19}$F retention index and $pO_2$ were compared ($r \leq 0.3$). The inability to observe a strong correlation between the two techniques was not altogether surprising as by using oxygen needle electrodes, low $pO_2$ values may also result from regions within the tumour that do not contain viable hypoxic cells, such as necrotic regions, whereas 2-nitroimidazoles label only hypoxic cells (Chapman et al, 1981; Lord et al, 1993; Aboagye et al, 1995*a*). This may also account for the higher variability of $pO_2$ values at low $^{19}$F retention indices as the influence of necrosis on the average $pO_2$ values obtained is potentially high. Despite the lack of overall correlation noted above, the study indicated that all tumours that showed substantial retention of $^{19}$F signal ($^{19}$F retention index of > 0.5) also had a high level of tumour hypoxia or low tumour oxygenation (% $pO_2 \leq 5$ mmHg = 60%). This observation is probably a result of the selective reductive activation of SR-4554 under such hypoxic conditions. Cytotoxicity of the chemical probe towards hypoxic cells is unlikely to contribute significantly to tumour oxygenation because nitroimidazoles such as SR-4554, which do not incorporate a cytotoxic side-chain, have very low cytotoxicity (Chapman et al, 1983). Previous studies in C3H mammary foot tumours suggested that when the percentage of $pO_2$ values $\leq$ 5 mmHg was 60% the equivalent clonogenic radiobiological hypoxic fraction was 10% (Nordsmark et al, 1995). We might, therefore, speculate that substantial trapping of the $^{19}$F signal could be evident in tumours with a radiobiological hypoxic fraction of > 10%. This level of sensitivity is likely to be of clinical relevance; for example, the radiocurability of human tumours is reduced with the presence of $\geq$ 26% of tumour cells with $pO_2 \leq 8$ mmHg (Gatenby et al, 1988).

As part of the present study, it was necessary to investigate the effect of anaesthesia on $pO_2$ as this form of restraint may be required in experimental studies with SR-4554. Oxygen tension measurements indicated that the anaesthetic used (Hypnorm–Hypnovel–water) did not significantly alter tumour oxygenation in the C3H mammary tumour model. This is consistent with our $^{31}$P bioenergetic measurements (Nordsmark et al, 1995; M Nordsmark et al, submitted for publication). Sansom and Wood (1994) have also demonstrated the lack of significant $^{31}$P spectral changes in anaesthetized (Hypnorm–Hypnovel–water) vs tumours in conscious mice. These are very interesting findings as other widely used anaesthetics, such as isoflurane and halothane, have been shown to alter tumour characteristics including blood flow and bioenergetics (Zhao et al, 1995). In contrast, however, SR-4554 alone and the combination of SR-4554 and anaesthetic produced a small but significant increase in tumour oxygenation when medians or the percentage of $pO_2$ values $\leq$ 2.5 mmHg were compared. It is unknown whether this effect of SR-4554 is due to a direct effect on the oxygen electrode, e.g. by an electrochemical effect, or specifically due to the effect of the compound on tumour oxygenation. This interesting observation is, however, contrary to the decrease in tumour blood flow (and hence oxygenation) produced by another 2-nitroimidazole, pimonidazole, at a dose of 500 mg kg$^{-1}$ body weight in the same tumour model measured over 1 h (Chaplin and Horsman, 1992). Provided the same anaesthesia/SR-4554 protocol is used in all experimental animal studies, however, this increase in oxygenation produced by the combination of SR-4554 and anaesthesia will not be expected to affect the use of SR-4554 to measure tumour oxygenation. It is, nevertheless, an aspect that should be investigated in any clinical trials with SR-4554.

An obvious concern in the design of the present experiments was whether the measurement of $pO_2$ in SR-4554 treated tumours will alter the $^{19}$F data determined by MRS. Importantly, no significant differences in $^{19}$F retention were observed between tumours that had their $pO_2$ measured and those that did not (Kruskal–Wallis test; 95% confidence level), indicating that the $pO_2$ measurements by themselves did not significantly affect the retention of SR-4554.

An important consideration, which is relevant to the clinical applicability of this $^{19}$F MRS method, is signal sensitivity. The dose of drug used in this study (180 mg kg$^{-1}$) is non-toxic (Aboagye et al, 1996), and up to 1300 mg kg$^{-1}$ can be safely administered without any observable toxic effect in non-tumour-bearing female Balb/c mice. This implies that the sensitivity of detection can be further enhanced by the administration of higher doses. With regard to signal sensitivity per mole of $^{19}$F, it is worth mentioning that, although these experiments were carried out at 7 tesla, the compound is easily detectable at the same dose at 4.7 tesla. Similar experiments with the monofluorinated 2-nitroimidazole Ro 07-0741 at 1.9 tesla suggests that SR-4554 will be detected using clinical MR instruments that are available currently (1.5–4 tesla). Considering the feasibility of these methods in patients, it should be noted that, even if magnetic field strength and drug dosage were lower, tumour volume can be substantially greater than in the murine tumours studied here.

In summary, the present studies have shown that the fluorinated 2-nitroimidazole SR-4554 has the ability to detect clinically relevant levels of tumour hypoxia and have provided useful information for the clinical development of SR-4554 as a non-invasive probe for use in man. No single method is necessarily ideal for measuring therapeutically relevant tumour hypoxia, as all have potential advantages and disadvantages. The important requirement is to obtain information on the correlation with clinical outcome in man.

### ACKNOWLEDGEMENTS

The authors would like to acknowledge financial support of the Cancer Research Campaign (UK), Karen Elise Jensens Fund (Denmark), Danish Cancer Society and Overseas Research Scholarship (awarded to EOA). PW acknowledges the award of a CRC Life Fellowship.

### REFERENCES

Aboagye EO, Lewis AD, Johnson A, Workman P, Tracy M and Huxham IM (1995*a*) The novel fluorinated 2-nitroimidazole hypoxia probe SR-4554: reductive metabolism and semi-quantitative localization in human ovarian cancer multicellular spheroids as measured by electron energy loss spectroscopic analysis. *Br J Cancer* **72:** 312–318

Aboagye EO, Graham MA, Lewis AD, Workman P, Kelson AB and Tracy M (1995*b*) Development and validation of a solid-phase extraction and high-performance liquid chromatographic assay for a novel fluorinated 2-nitroimidazole hypoxia probe (SR-4554) in Balb/c mouse plasma. *J Chromatogr B* **672:** 125–132

Aboagye EO, Lewis AD, Graham MA, Tracy M, Kelson AB, Ryan KJ & Workman P (1996) The pharmacokinetics, bioavailability, and biodistribution of a rationally designed 2-nitroimidazole hypoxia probe SR-4554. *Anti-Cancer Drug Des* **11:** 231–242

Aboagye EO, Maxwell RJ, Kelson AB, Tracy M, Lewis AD, Graham MA, Horsman MR, Griffiths JR and Workman P (1997) Preclinical evaluation of the

© Cancer Research Campaign 1998

fluorinated 2-Nitroimidazole *N*-(2-hydroxy-3,3,3-trifluoropropyl)-2-(2-nitro-1-imidazolyl) acetamide (SR-4554) as a probe for the measurement of tumor hypoxia. *Cancer Res* **57**: 3314–3318

Brizel DM, Rosner GL, Prosnitz LR and Dewhirst MW (1995) Patterns and variability of tumor oxygenation in human soft tissue sarcomas, cervical carcinomas and lymph node metastases. *Int J Radiat Oncol Biol Phys* **32**: 1121–1125

Chaplin DJ and Horsman MR (1992) Tumour blood flow changes induced by chemical modifiers of radiation response. *Int J Radiat Oncol Biol Phys* **22**: 459–462

Chapman JD (1984) The detection and measurement of hypoxic cells in solid tumours. *Cancer* **54**: 2441–2449

Chapman JD, Franko AJ and Sharplin J (1981) A marker for hypoxic cells in tumors with potential clinical applicability. *Br J Cancer* **43**: 546–550

Chapman JD, Baer K and Lee J (1983) Characteristics of the metabolism-induced binding of misonidazole to hypoxic mammalian cells. *Cancer Res* **43**: 1523–1528

Franko AJ, Koch CJ, Garrecht BM, Sharplin J & Hughes D (1987) Oxygen dependence of binding of misonidazole to rodent and human tumours *in vitro*. *Cancer Res* **47**: 5367–5376

Gatenby RA, Kessler HB, Rosenblum JS, Coia LR, Moldofsky PJ, Hartz WH and Broder GJ (1988) Oxygen distribution in squamous cell carcinoma metastases and its relationship to outcome of radiation therapy. *Int J Radiat Oncol Biol Phys* **14**: 831–838

Horsman MR, Khalil AA, Siemann DW, Grau C, Hill SA, Lynch E, Chaplin DJ and Overgaard J (1994) Relationship between radiobiological hypoxia in tumours and electrode measurements of tumour oxygenation. *Int J Radiat Oncol Biol Phys* **29**: 439–442

Jin G-Y, Li S-J, Moulder JE and Raleigh JA (1990) Dynamic measurements of hexafluoromisonidazole (CCI-103F) retention in mouse tumours by $^{1}H/^{19}F$ magnetic resonance spectroscopy. *Int J Radiat Biol* **58**: 1025–1034

Joseph P, Jaiswal AK, Stobbe CC and Chapman JD (1994) The role of specific reductases in the intracellular activation and binding of 2-nitroimidazoles. *Int J Radiat Oncol Biol Phys* **29**: 351–355

Kavanagh MC, Sun A, Hu Q and Hill RP (1996) Comparing techniques of measuring tumour hypoxia in different murine tumors: Eppendorf $pO_2$ Histograph, [$^{3}$H]misonidazole binding and paired survival assay. *Radiat Res* **145**: 491–500

Koch CJ, Stobbe CC and Baer KA (1984) Metabolism induced binding of $^{14}$C-misonidazole to hypoxic cells: kinetic dependence on oxygen concentration and misonidazole concentration. *Int J Radiat Oncol Biol Phys* **10**: 1327–1332

Kolstad P (1968) Intercapillary distance, oxygen tension and local recurrence in cervix cancer. *Scand J Clin Lab Invest* **22** (suppl. 106): 145–157

Kwock L, Gill M, McMurry HL, Beckman W, Raleigh JA and Joseph AP (1992) Evaluation of a fluorinated 2-nitroimidazole binding to hypoxic cells in tumour-bearing rats by $^{19}$F magnetic resonance spectroscopy and immunohistochemistry. *Radiat Res* **129**: 71–78

Lord EM, Harwell L and Koch CJ (1993) Detection of hypoxic cells by monoclonal antibody recognizing 2-nitroimidazole adducts. *Cancer Res* **53**: 5721–5726

Maxwell RJ, Workman P, Griffiths JR (1988) Demonstration of tumour-selective retention of fluorinated probed by $^{19}$F magnetic resonance spectroscopy in vivo. *Int J Radiat Oncol Biol Phys* **16**: 925–929

Mueller-Klieser W, Schlenger KH, Walenta S, Gross M, Karbach U, Hoeckel M and Vaupel P (1991) Pathophysiological approaches to identifying tumor hypoxia in patients. *Radiother Oncol* **20** (suppl. 1): 21–28

Nordsmark M, Grau C, Horsman MR, Jorgensen HS and Overgaard J (1995). Relationship between tumour oxygenation, bioenergetic status and radiobiological hypoxia in an experimental model. *Acta Oncol* **34**: 329–334

Okunieff P, Hoeckel M, Dunphy EP, Schlenger K, Knoop C and Vaupel P (1993) Oxygen tension distributions are sufficient to explain the local response of human breast tumors treated with radiation alone. *Int J Radiat Oncol Biol Phys* **26**: 631–636

Olive PL and Durand RE (1989) Misonidazole binding in SCCVII tumours in relation to the tumour blood supply. *Int J Radiat Oncol Biol Phys* **16**: 755–761

Overgaard J (1980) Effect of misonidazole and hyperthermia on the radiosensitivity of a C3H mouse mammary carcinoma and its surrounding normal tissue. *Br J Cancer* **41**: 10–21

Raleigh JA, Franko AJ, Kelly DA, Trimble LA and Allen PS (1991) Development of an in vivo $^{19}$F magnetic resonance method for measuring oxygen deficiency in tumours. *Magn Reson Med* **22**: 451–466

Rampling R, Cruickshank G, Lewis AD, Fitzsimmons S and Workman P (1994) Direct measurement of $pO_2$ distribution and bioreductive enzymes in human malignant brain tumours. *Int J Radiat Oncol Biol Phys* **29**: 427–431

Sansom JM and Wood PJ (1994) $^{31}$P MRS of tumour metabolism in anesthetized vs conscious mice. *NMR Biomed* **7**: 167–171

Twentyman PR, Brown MJ, Gray JW, Franko AJ, Scoles MA and Kallman RF (1980) A new mouse tumour model system (RIF-1) for comparison of end-point studies. *J Natl Cancer Inst* **64**: 595–604

Vaupel P, Schlenger K, Knoop C and Hockel M (1991) Oxygenation of human tumours: Evaluation of tissue oxygen distribution in breast cancers by computerized $O_2$ tension measurements. *Cancer Res* **51**: 3316–3322

Vaupel P, Schlenger KH, Hoeckel M and Okunieff P (1992) Oxygenation of mammary tumors: from isotransplanted rodent tumors to primary malignancies in patients. *Adv Exp Med Biol* **316**: 361–371

Zhao M, Fortan LG and Evelhoch JL (1995) The effects of isoflurane and halothane on blood flow and $^{31}$P NMR spectra in murine RIF-1 tumours. *Magn Reson Med* **33**: 610–618

© Cancer Research Campaign 1998